# Abemaciclib, Palbociclib, and Ribociclib in Real-World Data: A Direct Comparison of First-Line Treatment for Endocrine-Receptor-Positive Metastatic Breast Cancer

**DOI:** 10.3390/ijms24108488

**Published:** 2023-05-09

**Authors:** Mónica Cejuela, Ana Gil-Torralvo, M. Ángeles Castilla, M. Ángeles Domínguez-Cejudo, Alejandro Falcón, Marta Benavent, Sonia Molina-Pinelo, Manuel Ruiz-Borrego, Javier Salvador Bofill

**Affiliations:** 1 Medical Oncology Department, Virgen del Rocio Hospital, 41013 Seville, Spainafalconglez@gmail.com (A.F.);; 2 Institute of Biomedicine of Seville (IBiS), HUVR, CSIC, Universidad de Sevilla, 41013 Seville, Spain; 3 Andalusia-Roche Network Mixed Alliance in Precision Medical Oncology, 41092 Sevilla, Spain

**Keywords:** CDK4/6 inhibitors, cyclin inhibitors, breast cancer, breast carcinoma, metastatic, endocrine therapy, abemaciclib, palbociclib, ribociclib, real world

## Abstract

By the end of 2020, there were more than 8 million women alive who had received a breast cancer diagnosis in the previous 5 years, making it the most prevalent neoplasia in the world. About 70% of breast-cancer cases present positivity for estrogen and/or progesterone receptors and a lack of HER-2 overexpression. Endocrine therapy has traditionally been the standard of care for ER-positive and HER-2-negative metastatic breast cancer. In the last 8 years, the advent of CDK4/6 inhibitors has shown that adding them to endocrine therapy doubles PFS. As a result, this combination has become the gold standard in this setting. Three CDK4/6 inhibitors have been approved by the EMA and the FDA: abemaciclib, palbociclib, and ribociclib. They all have the same indications, and it is at each physician’s discretion to choose one or the other. The aim of our study was to perform a comparative efficacy analysis of the three CDK4/6i using real-world data. We selected patients diagnosed with endocrine-receptor-positive and HER2-negative breast cancer who were treated with all three CDK4/6i as first-line therapy at a reference center. After 42 months of retrospective follow up, abemaciclib was associated with a significant benefit in terms of progression-free survival in endocrine-resistant patients and in the population without visceral involvement. In our real-world cohort, we found no other statistically significant differences among the three CDK4/6 inhibitors.

## 1. Introduction

Breast cancer is the most common neoplasia worldwide and is the leading cause of women’s mortality due to cancer [1]. About 70% of breast cancer cases present positivity for estrogen and/or progesterone receptors in the absence of human epidermal growth factor receptor 2 (HER-2) overexpression. This group is considered to respond to endocrine therapy (ET) and has a better prognosis, with long survival rates even in a metastatic setting [2].Especially, for those patients with less aggressive tumors and without visceral disease [3,4]. Nevertheless, almost all of these patients will develop endocrine resistance in their lifetime [5].

Cyclin-dependent kinase 4 and 6 (CDK4/6) and retinoblastoma (Rb) deregulation play a key role in endocrine resistance. CDK4/6 inhibitors (CDK4/6i) block the activity of the cyclin D1-CDK4/6 holoenzyme and subsequent dephosphorylation of the Rb tumor suppressor. This results in the blockage of cell-cycle progression from G1 to S phase [6,7]. The inhibition of estrogen signaling pathways has shown similar effects in reducing CDK4 and CDK6 activation. This supports the rationale for the combination of CDK4/6i with ET [8]. There are currently three CDK4/6i that have been approved by the European Medicines Agency (EMA) and the Food and Drug Administration (FDA) for metastatic endocrine-receptor (ER)-positive and HER-2 negative breast cancer in combination with aromatase inhibitors (AI) or fulvestrant, namely: abemaciclib, palbociclib, and ribociclib. Table 1 summarizes the main studies and the data for their approval. Palbociclib was the first CDK4/6i to receive FDA approval in February 2015. It first became indicated in combination with AI following the results of the PALOMA 1 and PALOMA 2 studies [9,10]. Later, the indication was expanded to its combination with fulvestrant (PALOMA 3 trial) [11]. Ribociclib first received FDA approval in March 2017. It was supported by the MONALEESA program, where it demonstrated benefits both in progression-free survival (PFS) and in overall survival (OS) [12,13,14]. By September 2017, the results of the MONARCH-2 trial provided abemaciclib accelerated approval in combination with fulvestrant [15]. The MONARCH-3 trial showed that abemaciclib was also effective when combined with AI [16]. In addition, abemaciclib has been shown to be beneficial as a monotherapy in heavily pretreated patients (MONARCH-1 study) [17]. On the other hand, abemaciclib is the only CDK4/6i approved for adjuvant use in high-risk ER-positive and HER2-negative patients [18].

Although the three drugs act on the cyclin pathway, they seem to differ in important ways. Firstly, not all CDK4/6i inhibit CDKs with equal potency. CDK4 and CDK6 inhibition with half maximal inhibitory concentrations are 11 nM and 15 nM (CDK4:CDK6 ratio of 1:1.5) for palbociclib, 10 nM and 39 nM (1:4) for ribociclib, and 2 nM and 10 nM (1:5), for abemaciclib, respectively [19,20]. Palbociclib and ribociclib seem to be more selective for CDK4/6, with less engagement against the rest of CDK. Abemaciclib also inhibits CDK1/2/9/16/17 with potencies comparable to those for CDK4/6, and is a weaker inhibitor of CDK5/7/8/12/13/14/15/19 [21]. The ability of abemaciclib to inhibit other cyclins makes it effective at inhibiting cell growth, even in cell lines with Rb deficiency. In addition to inducing cell-cycle arrest in G1, abemaciclib also appears to suppress the cell cycle in G2 and even cause tumor regression in preclinical models [22,23]. These properties would be supported by the fact that it is the only cyclin that has shown clinical benefit as a monotherapy [18]. Potent targeting of CDK9 could justify the intestinal toxicity observed with abemaciclib, whereas the stronger blockage on CDK6 would explain the higher hematological toxicity for palbociclib and ribociclib. Disparities in pharmacokinetics have also been described [19,20,24]. Studies have displayed saturation of drug absorption only for abemaciclib, which supports the twice-daily dosage. The three CDK4/6i are metabolized hepatically, primarily mediated by CYP3A4. Palbociclib is also metabolized in the small intestine and adrenal cortex. Palbociclib and ribociclib have moderate binding to human plasma proteins and a high volume of distribution, whereas abemaciclib is highly bound to the plasma proteins [19,20,24]. Abemaciclib can better penetrate mammary tissue and the blood−brain barrier due to its improved lipophilicity. It has even been shown to reduce tumor growth in the brain. On the contrary, both palbociclib and ribociclib are substrates of breast cancer resistance protein and P-glycoprotein, which may limit their ability to penetrate the central nervous system [19,25].

Whether these differences may have an impact on their antitumor activity is not yet known. Despite similar results in terms of PFS, ribociclib still is the CDK4/6i with the strongest evidence of OS benefit. The choice of one or the other CDK4/6i remains largely determined by the patient and their toxicity profile, potential drug interactions, and the physician’s own experience. We still do not have study results that directly compare these drugs. At present, there is only one ongoing phase III trial comparing-face to-face ribociclib and palbociclib (NCT05207709). However, this trial mainly focuses on the HER2-enriched population. The aim of our study is to analyze the evolution of patients treated with the different CDK4/6i as front-line therapy to determine whether there are significant differences in patient outcome in our real-world data population.

## 2. Results

### 2.1. Patients Characteristics

A total of 206 patients were identified and retrospectively followed for 42 months. Median follow-up was 27.64 months (IQR: 1.31–42 months). Complete information about baseline and clinical patient characteristics is summarized in Table 2. Median age at the time of CDK4/6i initiation was 60.9 years. All patients were women, and most were in menopause (80.6%). Out of all patients, 56 (27.2%) had de novo metastatic disease, while 150 (72.8%) had recurrences. Upon review of disease extension, 116 patients (56.3%) had visceral metastases and 60 (29.1%) bone-only disease; 78 patients had luminal A-like phenotype (37.9%), while 114 presented luminal-B-like phenotype (55.3%). Of the studied population, 78 patients (37.9%) were considered endocrine resistant: 20 patients (25.6%) had primary resistance and 58 (74.4%) had secondary resistance. Clinicopathologic characteristics were similarly distributed (*p* > 0.05) among the three treatment arms, except for age at treatment initiation and menopausal status.

### 2.2. Exposure to CDK4/6 Inhibitors

Abemaciclib was administered to 56 (27.2%) patients, 96 (46.6%) palbociclib, and 54 (26.3%) ribociclib. Almost half of the patients (43.7%) were prescribed fulvestrant and 116 (56.3%) AI. Most patients started treatment on a full dose schedule. However, 7 patients began treatment with dose adjustments: 5 with palbociclib, 1 with abemaciclib, and 1 with ribociclib. CDK4/6i dose reductions were required in 108 of the patients analyzed (52.4%), 53.6% in patients treated with abemaciclib, 52.1% in patients treated with palbociclib, and 51.9% in patients treated with ribociclib. Almost all patients discontinued treatment due to disease progression, with the exception of three patients who discontinued due to treatment toxicity and two patients who discontinued ribociclib due to toxicity (one patient discontinued due to hepatic and hematologic toxicity and the other patient discontinued due to cutaneous toxicity). The remaining patient discontinued abemaciclib due to hepatic toxicity.

### 2.3. Clinical Outcomes in the Overall Population

To retrospectively assess the benefit of each CDK4/6i in our population, we evaluated PFS in the general population and with each CDK4/6i. Based on RECIST 1.1 criteria and using scans performed according to standard clinical practice (3–6 months), we found an overall PFS of 35.61 months. Using a univariate logistic regression model, patients treated with palbociclib had a 2.57-fold increased risk of progression compared with patients treated with abemaciclib (odds ratio (OR) = 2.57; 95% confidence interval (CI), 1.14–5.79, *p* = 0.022), while patients treated with ribociclib had a 3.37-fold increased risk of progression compared with patients treated with abemaciclib (OR = 3.37; 95% CI, 1.37–8.31; *p* = 0.008). The overall response rate (ORR) was 45.1%. Most patients achieved disease stabilization (42.7%) or a partial response (34.5%) to CDK4/6i. Specifically, 21 (37.5%), 48 (50%), and 19 (35.2%) patients achieved stabilization, while 26 (46.4%), 27 (28.1%), and 18 (33.3%) patients achieved a partial response to abemaciclib, palbociclib, and ribociclib, respectively. Moreover, 5 (9.9%), 8 (8.3%), and 9 (16.7%) patients achieved a complete response, respectively. We found an ORR of 57.5% (23) in premenopausal patients: 5 patients achieved complete response and 18 achieved partial responses. Fourteen premenopausal women achieved disease stabilization. Table 3 summarizes treatment exposure data and patient response for each CDK4/6i.

**Figure 1:**
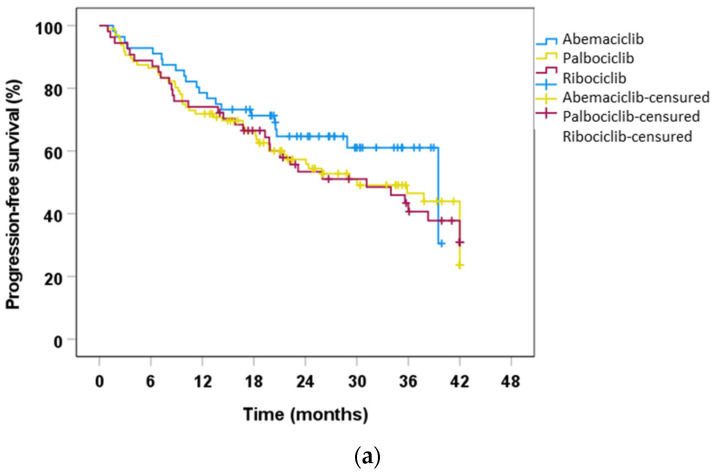

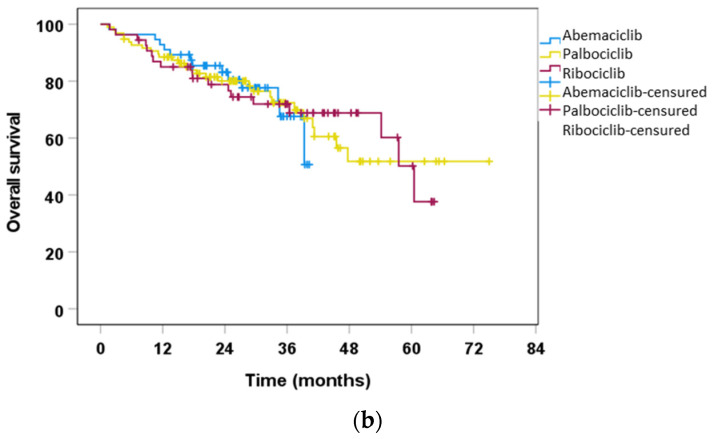
(**a**) Progression-free survival according to CDK4/6i. Kaplan−Meier analysis of PFS comparing the different CDK4/6i in the study population. Abemaciclib−palbociclib, *p* = 0.241, by Breslow test; abemaciclib−ribociclib, *p* = 0.270, by Breslow test; palbociclib−ribociclib, *p* = 0.984, by Breslow test. (**b**) Overall survival according to CDK4/6i. Kaplan−Meier analysis of OS. Abemaciclib−Palbociclib, *p* = 0.791, by Breslow test; abemaciclib−ribociclib, *p* = 0.659, by Breslow test; palbociclib−ribociclib, *p* = 0.904, Breslow test.

Follow up was much shorter for abemaciclib (40.28 months vs. 75 months for palbociclib and 64.4 months for ribociclib) because it was the last drug to be approved. Therefore, we used a cut-off time of 42 months for the PFS and OS analyses to compare them. PFS was 39.49 months with abemaciclib, 30.03 months with palbociclib, and 31.14 months with ribociclib (Figure 1a). These were not statistically significant differences when using the Kaplan−Meier test. Regarding dose reductions, we found no significant differences among the three CDK4/6i in PFS in patients who required CDK4/6i dose adjustments. In these patients, the median PFS for palbociclib was 35.87 months and 36.01 months for ribociclib. The median PFS for abemaciclib was not reached at the cutoff. After the established follow-up period, 109 (52.9%) patients were still on CDK4/6i treatment and 97 (47.1%) patients had progressed, 60 (29.1%) of whom had died. Among the patients who died, 14 of them (25.5%) received abemaciclib, 26 (47.3%) palbociclib, and 15 (27.3%) ribociclib. Concerning OS, the whole study population presented an OS of 57.56 (95% CI, 44.59–70.52 months). In the Kaplan−Meier analysis performed, we found no significant differences in OS among the three inhibitors, and the median was not reached by any of the three drugs after 42 months of follow up (Figure 1b).

### 2.4. Clinical Outcomes in the Endocrine Resistant Population

**Figure 2:**
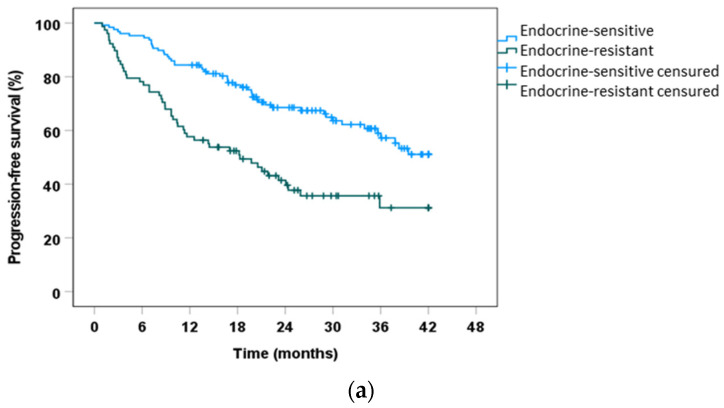

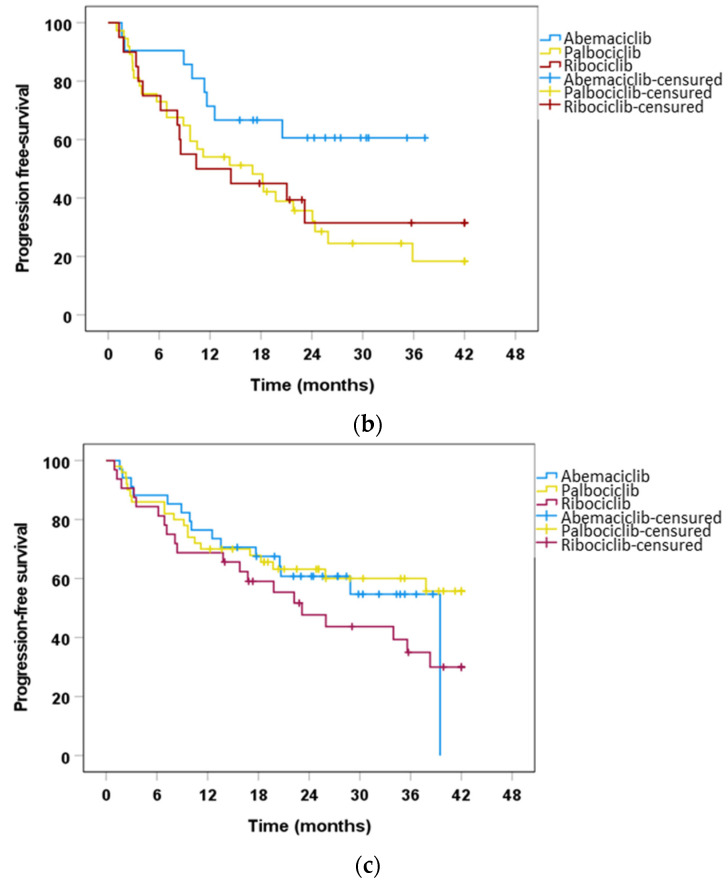
Progression-free survival according to endocrine resistance. (**a**) Kaplan-Meier analysis of PFS comparing endocrine-sensitive patients to endocrine-resistant patients, *p* < 0.001 by log rank test. (**b**) Kaplan-Meier analysis of PFS comparing the different CDK4/6i in patients with endocrine resistance. Abemaciclib−palbociclib, *p* = 0.027, by log rank test; abemaciclib−ribociclib, *p* = 0.070 by log rank test; palbociclib−ribociclib, *p* = 0.972, by Breslow test. (**c**) Kaplan−Meier analysis of PFS comparing the different CDK4/6i in endocrine-sensitive patients. Abemaciclib−palbociclib: *p* = 0.726 by Breslow test; abemaciclib-ribociclib: *p* = 0.936 by Breslow test; palbociclib−ribociclib: *p* = 0.629 by Breslow test.

Endocrine resistance has been shown to be associated with a worse prognosis. To determine whether this affected the outcome of our patients, we evaluated PFS in the endocrine-resistant population, which was found to be 18.30 months. In contrast, median PFS was not achieved in the endocrine-sensitive population (*p* < 0.001). An additional objective was to assess whether any CDK4/6i would be more beneficial in patients considered to be endocrine resistant. In this population, abemaciclib showed better PFS compared with palbociclib (not achieved vs. 17.02 months; *p* < 0.05). Nevertheless, no statistically significant difference was found in PFS when comparing abemaciclib to ribociclib (10.38 months, *p* = 0.07) and palbociclib to ribociclib (*p* = 0.972). Hazard ratios for PFS in the endocrine-sensitive population were 2.41 (*p* = 0.029; 95% CI, 1.09–5.31) for palbociclib and 2.19 (*p* = 0.081; 95% CI, 0.91–5.31) for ribociclib compared with abemaciclib in univariate Cox proportional hazards regression analysis.Figure 2 shows the PFS curves of patients according to their endocrine resistance status.

### 2.5. Clinical Outcomes in the Population with Visceral Disease

**Figure 3:**
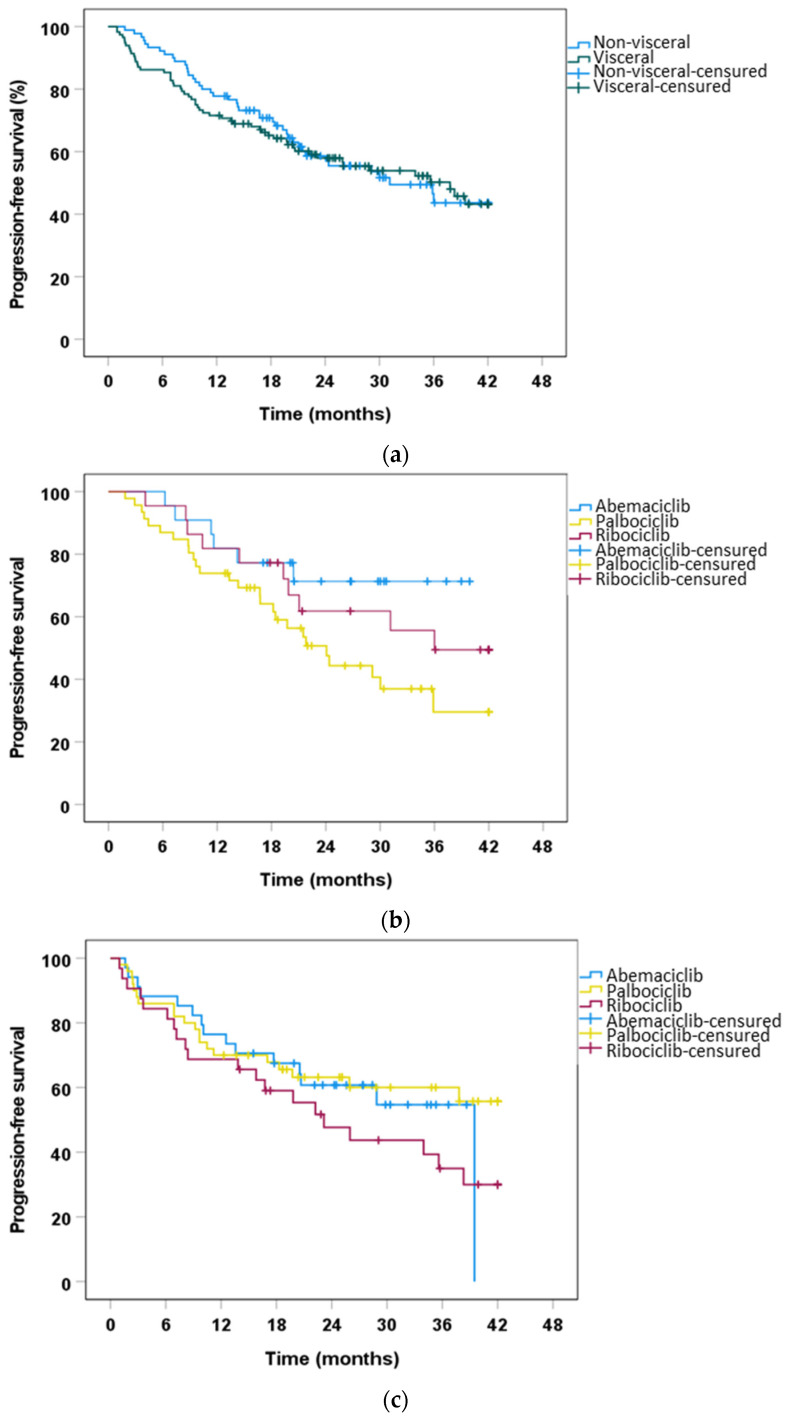
Kaplan-Meier analysis of progression-free survival benefit of the three CDK4/6i in visceral and non-visceral disease. (**a**) Kaplan−Meier analysis of PFS comparing the non-visceral vs. visceral population, *p* = 0.469, by Breslow test. (**b**) Kaplan−Meier analysis of PFS comparing the three CDK4/6i in the population without visceral involvement. Abemaciclib−palbociclib, *p* = 0.038, by log rank test; abemaciclib−ribociclib, *p* = 0.581, by Breslow test; palbociclib−ribociclib, *p* = 0.163, by log rank test. (**c**) Kaplan−Meier analysis of PFS comparing the three CDK4/6i in the population with visceral disease. Abemaciclib−palbociclib, *p* = 0.937, by Breslow test; abemaciclib−ribociclib, *p* = 0.307, by Breslow test; palbociclib−ribociclib, *p* = 0.255, by Breslow test.

When we stratified patients by disease extension, the estimated median PFS was 31.14 months (95% CI, 19.61–42.67) for non-visceral disease and 37.81 months (95% CI, 25.93–49.70) for visceral disease (*p* > 0.05). In the subgroup of patients without visceral involvement, median PFS was significantly longer in the abemaciclib group (not reached) compared with the palbociclib (24.1 months, 95% CI, 18.04–30.12; *p* = 0.038) and ribociclib (36.01 months). In the subgroup of patients with visceral involvement, there were no significant differences in PFS among the three drugs (abemaciclib 39.49 months vs. palbociclib not reached vs. ribociclib 23.16 months; 95% CI; 13.58–32.74). Figure 3 shows PFS curves for patients with visceral and non-visceral disease. 

## 3. Discussion

Prescribing abemaciclib, palbociclib, and ribociclib as front-line therapy showed differences in the outcome of patients with metastatic breast cancer in our real-world data population. To the best of our knowledge, this is the biggest comparative analysis among the three CDK4/6i in a retrospective cohort. As such, it gives insight into the efficacy and safety of CDK4/6i in a broad population of patients. In our study, despite being a retrospective analysis, most of the patient characteristics were well balanced among the three treatment arms. The mean age was 60 years, which is consistent with the prospective studies (57–63 years). In addition, some of the main clinical characteristics, such as the percentage of the novo metastatic disease (27.2% in our cohort vs. 20–41%), the proportion of patients with visceral involvement (56.3% vs. 48–61%), and bone-only disease (29.1% vs. 21–28%) were consistent with those published in the drug registration studies

Patients were mainly prescribed palbociclib (46.6%) because it was the first to receive approval and commercialization in our country. We consider that the high percentage of patients receiving fulvestrant as first-line therapy (43.7%) may be related to the results of the FALCON study, which showed that fulvestrant provided longer PFS compared with AI as first-line endocrine therapy [26]. However, following PARSIFAL data, letrozole remains the preferred endocrine therapy for patients without endocrine resistance [27]. Younger age is associated with more aggressive tumor biology, higher grade, and more advanced disease, and these patients tend to have a worse prognosis [28,29]. In the premenopausal population of our study, almost half (48%) of the patients received ribociclib. This may be due to the results of the MONALEESA 7 study, which included 672 premenopausal women [14], whereas premenopausal women were less represented in the MONARCH 2 and PALOMA 3 studies, with 17% *(N* =114) and 21% (*N* =108) of the total population, respectively [15,30]. In these three studies, premenopausal women showed a similar benefit to the postmenopausal population. In our analysis, the ORR achieved in premenopausal women was higher than in the general population, with ribociclib being the CDK4/6i with the highest ORR. However, our cohort of premenopausal patients was small and poorly balanced, so no conclusions could be drawn from these data. 

CDK4/6i are generally well tolerated, but it is estimated that up to half of those patients require dose reductions at some point. Dose reductions were reported in 43% of patients in the MONARCH 2 and MONARCH 3 safety analyses, which did not appear to affect PFS [31]. Analysis from the MONALEESA program also showed that 41.7% of patients required at least one dose reduction without affecting tumor outcome [32]. For palbociclib, an estimated 35% of the palbociclib trial population required dose reductions [33]. Real-world CDK4/6i data are emerging. In line with some of the published data [34], we found that 52.4% of our patients required treatment changes, which were more frequent in patients older than 70 years (68%). The proportion of dose reductions were similar in the three CDK4/6i arms. The differences seen in the real-world data compared to pivotal trials could be related to the inclusion of fewer eligible patients and a more individualized management of toxicity. We found a longer PFS with 37.81 months after dose reduction compared with 25.98 months (95% CI, 12.78–39.79) when patients received the initial dose (*p* < 0.05). Although these data could appear inconsistent, other real-world series have shown similar results [35,36]. This may be related to longer treatment maintenance in patients with better tolerability after dose adjustment. Another hypothesis could be that dose-reduced patients would require fewer treatment interruptions, resulting in more stable plasma drug levels and the avoidance of tumor hyperactivation.

The median PFS in our population receiving ribociclib was 31.14 months (95% CI, 15.78–46.52). These results were compared to the PFS achieved in the ribociclib trials including both endocrine-resistant and -sensitive patients. In MONALEESA 3, the median PFS achieved with ribociclib in the first-line treatment cohort was 33.6 months (95% CI, 27.1–41.3), while in MONALEESA 7 the PFS was 23.8 months (95% CI, 19.2, not reached) [13,37]. However, it should be noted that MONALEESA 7 included only premenopausal patients and up to 70% of patients had disease recurrence compared with 59% in our population [37]. Our PFS for palbociclib was approximately 30 months (95% CI, 17.41–22.85). It was longer according to the results from the Spanish real-world data database of palbociclib (PALBOSPAIN) as first-line therapy, which showed a median PFS of 24 months (95% CI, 21–27) [34]. Although the proportion of postmenopausal patients was much lower in the PALBOSPAIN study (64.8% vs. 79% of our study population, respectively), the presence of visceral metastases (59.3% vs. 54.9%) and de novo metastatic disease (29.6% vs. 30.6%) were similar in both studies. Therefore, it is likely that these differences in PFS were also related to other clinical characteristics. Finally, there was a trend towards better PFS with abemaciclib (39.5 months, 95% CI, 24.58–54.4), but it did not reach statistical significance. Univariate logistic regression supported these results, with a two-fold lower risk of cyclin C progression in favor of abemaciclib compared with palbociclib and ribociclib.

In our cohort of endocrine-resistant patients, we observed that patients with abemaciclib achieved longer PFS than with the other two CDK4/6i. Abemaciclib did not achieve median PFS compared with 17 months with palbociclib (95% CI, 8.31–25.73) and 10.38 months in the ribociclib arm (95% CI, 0.00–23.34). We examined the percentage of patients diagnosed with relapse, the presence of visceral disease, elevated Ki67, and percentage of patients with luminal B-like phenotype in each CDK4/6i arm to exclude bias due to the presence of other poor prognostic factors. Notably, apart from a higher proportion of patients with ki67 ≥20% in patients with abemaciclib, all were well balanced across the three CDK4/6i. The median PFS observed for abemaciclib and palbociclib were higher than those observed in the pivotal trials in endocrine-resistant patients: MONARCH 2 (16.4 months) [15] and PALOMA 3 (11.2 months, 95% CI, 9.5 to 12.9) [11]. Given these findings, we looked for differences between MONARCH 2 and our population in some key clinical characteristics such as primary endocrine resistance (24.9% in MONARCH 2 vs. 33.3% in our population), visceral metastasis (54.9% vs. 60.7%), prior chemotherapy (59.9% vs. 52%), or premenopausal status (16.1% vs. 23.2%). Although PALOMA 3 included patients who had received prior treatment for metastatic breast cancer, who are considered to have a worse prognosis, we found no difference in premenopausal status and visceral disease between PALOMA 3 and our population (21% vs. 23% and 59% vs. 60%, respectively). Possible factors contributing to the observed numerically superior PFS may be influenced by other more favorable baseline characteristics in our patient population, and a more flexible approach to the re-evaluation of response in terms of study timing and assessment of clinical benefit compared with the strict windows for restaging scans in clinical trials. In contrast, our results for ribociclib were significantly shorter than those of the MONALEESA 3 study. In a recent MONALEESA 3 study analysis, the combination of ribociclib plus fulvestrant showed a PFS of 33.6 months (95% CI, 27.1–41.3 months) in first-line patients [13]. At the time of this report, we did not have the specific clinical characteristics of this patient subgroup, so we could not look for a direct justification among these data.

Approximately 56% of our cohort had visceral involvement. This rate is similar to the data published in the first-line cohort of the MONALEESA program (57.7%), PALOMA 2 (48.6%), PALOMA 3 (58.3%), MONARCH2 (55%), and MONARCH 3 (52%) [10,15,16,38,39]. The median PFS in our cohort with visceral extension was 37.81 months (95% CI, 25.93–49.70). In terms of PFS, our cohort showed similar results for ribociclib. Nevertheless, palbociclib and abemaciclib showed better PFS outcomes than in previously published data. In the population without visceral involvement, PFS was 31.14 months (95% CI, 19.61–42.67). In this subgroup, median PFS was significantly longer with abemaciclib than with palbociclib and ribociclib (not reached vs. 24.08 months, 95% CI, 18–30.12; *p* = 0.038 vs. 36.01 months, respectively).

Another controversial issue is whether these drugs are equally effective in prolonging OS. To date, only ribociclib has shown a statistically significant improvement in OS in all pivotal trials [13,14]. Data from MONARCH 2 showed that abemaciclib improved OS in patients with endocrine resistance [40]. However, MONARCH 3 showed that after a median follow-up of 5.8 years, abemaciclib plus AI prolonged OS (67.1 months), but did not reach statistical significance. Statistical significance is likely to be reached with a longer follow up. [41]. In PALOMA 2 and PALOMA 3, palbociclib did not show a statistically significant increase in OS [42,43]. Given that all three CDK4/6i have demonstrated a similar magnitude of benefit (HR) in terms of PFS, these results have been highly controversial. They were related to a significant loss of patients in the control arm and flaws in the study design for patient follow up. In our study population, we found an OS of 57.56 (95% CI, 44.59–70.52 months) at the time of the cutoff. At 42 months of median follow up, we did not find statistically significant differences between the three CDK4/6i.

After this follow up, the conclusions were robust but should not be considered definitive until more mature results are available, especially as the maximum follow-up time in patients treated with abemaciclib has not yet been reached. Given that the OS of a patient with metastatic breast cancer is approximately 60 months, longer follow up will also provide more conclusive information on the outcome.

In our routine practice, we follow the ESMO guidelines in metastatic breast cancer, with a choice between the three CDK4/6i depending on patient characteristics, potential comorbidities, and concomitant medications. Based on the MONALEESA-7 data mentioned above, there is a tendency to choose ribociclib in young patients. In patients with endocrine-resistant disease, there is a greater predisposition to abemaciclib based on the MONARCH 2 results, as well as in patients with central nervous system disease, where abemaciclib appears to have a higher penetrance. On the other hand, palbociclib would be a more attractive option in patients with drug−drug interactions, comorbidities, where it is particularly important to avoid diarrhea, or where poor tolerability is expected.

## 4. Materials and Methods

### 4.1. Study Design and Patient Population

**Figure 4:**
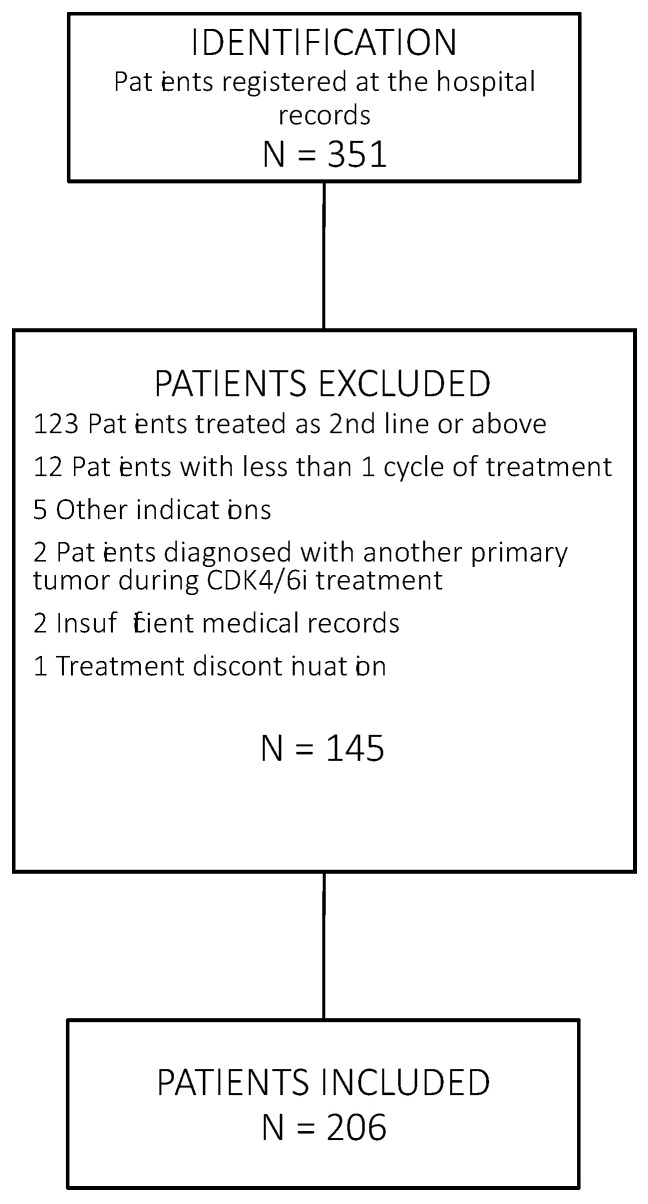
Flowchart of patient selection. The data source for this study was hospital pharmacy records. Patients were selected according to the inclusion criteria of this study: metastatic breast cancer patients receiving CDK4/6 inhibitors as first-line therapy.

A search was conducted on patients diagnosed with locally advanced or metastatic ER-positive and HER-2-negative breast cancer who were treated with CDK4/6i as a first-line therapy at the Hospital Virgen del Rocio between April 2014 and September 2021. Those who received at least one cycle of treatment with abemaciclib, palbociclib, or ribociclib plus ET and were followed up on for at least 1 year were selected. Patients who had received previous therapy for metastatic breast cancer were excluded. The patient selection procedure is shown in Figure 4. The selected population was monitored retrospectively. Endocrine resistance was established following the fourth ESO-ESMO International consensus recommendations. Primary endocrine resistance was defined as a relapse during the first 2 years of adjuvant ET, or disease progression within the first 6 months of first-line ET for metastatic breast cancer. Secondary endocrine resistance was defined as a relapse while on adjuvant ET, but after the first 2 years, or relapse within 12 months of completing adjuvant ET, or disease progression >6 months after initiating ET for metastatic breast cancer. Toxicity was graded according to the Common Terminology Criteria for Adverse Events (CTCAE). Treatment prescription and toxicity management were carried out according to the local product label. All premenopausal patients received parallel ovarian function suppression with luteinizing hormone-releasing hormone inhibitors, pelvic radiation double oophorectomy. Follow-up visits and tumor evaluations were carried out according to standard clinical practice. In general, patients were visited every 15 days for the first two months. Thereafter, visits were monthly until the sixth month, and then quarterly. Tumor assessments were carried out every 3–4 months. Those cases in which the disease was controlled after 2 years of treatment were seen every 4−6 months. ORR was defined as a patient proportion showing complete response or partial response over total patient population based on RECIST 1.1. Disease progression was determined by the recorded assessment of the attending clinician based on radiology, pathology, and clinical assessment. Data regarding histologic type, hormone receptors, Ki67 expression, and HER2 status were collected from pathology reports. Tumor stage and other clinical data, including treatment information, were obtained from electronic medical records. The study procedures were approved by the Coordinating Committee on Biomedical Research Ethics of Andalucía (0920-N20).

### 4.2. Statistical Analysis

Descriptive statistics were used to analyze each patient’s general characteristics (age, menopausal status, disease status, therapy, endocrine sensitivity, and metastatic locations) and tumor histopathological characteristics (endocrine receptor status, HER2 expression, and Ki67 proliferation index). Continuous variables were presented as the median values and interquartile range, and categorical variables were presented as percentages. To evaluate the statistical significance among the clinical variables of the patients who received the different treatments, categorical parameters were explored using the chi-squared test. Age at CDK4/6 initiation was a continuous variable with a normal distribution. Therefore, it was compared using the Anova test.

We established real-world PFS as the primary endpoint. Survival analyses were performed using the Kaplan−Meier method and compared with log-rank or Breslow tests. PFS was defined as the time in months from the start of treatment with CDK4/6i to the cutoff date, disease progression, or death. OS was defined as the time in months from starting CDK4/6i to death or last follow-up, whichever came first. Duration of follow-up was defined as the time in months from baseline to death or the data cutoff date of 1 October 2022, whichever occurred first. Hazard ratios and 95% confidence intervals for PFS were estimated from the Cox proportional hazards model. Two-sided *p*-values are presented for all analyses with *p* < 0.05 considered statistically significant. All statistical analyses were performed with SPSS 28.0 (Statistical Package for the Social Sciences).

## 5. Conclusions

To the best of our knowledge, our study is the largest population-based comparative analysis of the three CDK4/6i. Our results suggest that abemaciclib reduces the risk of disease progression more effectively when compared with ribociclib and palbociclib, demonstrating a benefit in progression-free survival in patients with refractory endocrine disease. Patients without visceral involvement also appear to benefit more from abemaciclib treatment. These data are based on a retrospective cohort from a single institution and should be considered with caution, as more evidence from prospective studies with larger sample sizes is needed to draw robust conclusions. Longer follow-up would allow us to see if these results are consistent and significant over time. Further work is needed to understand the biomarkers predicting benefit from each CDK4/6 inhibitor, to study the molecular changes induced, and to identify combinations that further improve patient prognosis.

## Figures and Tables

**Table 1:** Results from the pivotal phase III trials leading to the approval of abemaciclib, palbociclib, and ribociclib. All of them compared ET plus a CDK4/6 inhibitor or placebo.

CDK4/6i	Abemaciclib			Ribociclib		Palbociclib	
Trial (N)	MONARCH 2 672	MONARCH 3 493	MONALEESA -2 668	MONALEESA-3 726	MONALEESA-7 672	PALOMA 2 666	PALOMA 3 521
Combination ET	Fulvestrant	AI	Letrozole	Fulvestrant	Tamoxifen or AI plus GnRH	Letrozole	Fulvestrant (±GnRH)
Inclusion criteria	Pre/postmenopausal Endocrine resistant 2nd line ABC Prior ET allowed	Postmenopausal Endocrine sensitive 1st line ABC	Postmenopausal Endocrine sensitive 1st line ABC	Postmenopausal Endocrine sensitive/resistant 1st and 2nd line ABC Prior ET allowed	Premenopausal Endocrine sensitive/resistant 1st and 2nd line ABC No prior ET allowed, up to 1line of ChT	Postmenopausal Endocrine sensitive 1st line ABC	Endocrine resistant Pre/postmenopausal ≥2nd lines ABC
Population characteristics	Premenopausal: 16% Prior ET ABC: 38.3% Disease: Bone only: 27.6% Visceral: 55%	Disease: De novo: 41.2% Bone only: 21.3% Visceral: 52.4% Recurrence *: ≤36 months: 28% >36 months: 62.7%	Disease: De novo: 34 Bone only: 20.7% Visceral: 59% Recurrence *: ≤12 months: 1.2% >12 months: 64.7%	Prior lines ABC: 48.8% Disease: De novo: 20% Bone only: 21.3% Visceral: 60.5%	Prior Cht ABC: 14% Disease: De novo:41% Bone only: 24% Visceral: 58% Recurrence *: ≤12 months: 30% >12 months: 7%	Disease: De novo: 37% Bone only:23% Visceral: 48% Recurrence *: ≤12 months:22% >12 months: 40%	Premenopausal: 20.7% Prior ChT ABC: 30.8% Prior lines ABC: 0: 24.2%, 1: 38% 2: 26% ≥3: 11.8% Disease: Bone only: 24% Visceral: 59.4%
PFS in months ** and HR	16.4 vs. 9.3 HR: 0.553	29 vs. 14.8 HR: 0.518	25.3 vs. 16 HR: 0.568	20.5 vs. 12.8 HR: 0.593	23.8 vs. 13 HR: 0.55	27.6 vs. 14.5 HR: 0.56	11.2 vs. 4.6 HR: 0.497
OS in months *** and HR	46.7 vs. 37.3 HR: 0.757	67.1 vs. 54.5 HR: 0.754	63.9 vs. 51.4 HR: 0.76	53.7 vs. 41.5 HR: 0.73	58.7 vs. 48 HR: 0.76	53.9 vs. 51.2 HR: 0.956 95% CI, 0.777–1.177	34.9 vs. 28 HR: 0.81 95% CI, 0.64–1.03

* Recurrence is defined as the disease-free interval since the end of treatment. ** PFS of the study arm compared to the placebo arm. *** OS of the study arm compared to the placebo arm. Abbreviations: GnRH: gonadotropin-releasing hormone; ET: endocrine therapy; ABC: advanced breast cancer; Cht: chemotherapy; PFS: progression free survival; OS: overall survival; HR: hazard ratio.

**Table 2:** Clinicopathological characteristics of patients.

Characteristics	Total N (%)	Abemaciclib	Palbociclib	Ribociclib	*p*-Value
Gender (N)	206 female	56 (27.2%)	96 (46.6%)	54 (26.3%)	
Mean age *	60.9	60.6	63.1	57.3	0.018 ^a^
Menopausal status					
Premenopausal	40 (19.4%)	13 (23.2%)	8 (8.3%)	19 (35.2%)	0.0002 ^b^
Postmenopausal	166 (80.6%)	43 (76.8%)	88 (91.7%)	35 (64.8%)	
Concomitant ET					
Aromatase inhibitor Fulvestrant	116 (56.3%) 90 (43.7%)	29 (51.8%) 27 (48.2%)	53 (55.2%) 43 (44.8%)	34 (63%) 20 (37%)	0.476 ^b^
Disease status					
Stage IV at diagnosis	56 (27.2%)	15 (26.8%)	25 (26%)	16 (29.6%)	0.891 ^b^
Recurrence	150 (72.8%)	41 (73.2%)	71 (74%)	38 (70.4%)	
Metastasis extension					
Visceral	116 (56.3%)	34 (60.7%)	50 (52.1%)	32 (59.3%)	0.514 ^b^
Non-visceral	90 (43.7%)	22 (39.3%)	46 (47.9%)	22 (40.7%)	
Bone-only	60 (81.1%)	12 (70.6%)	33 (82.5%)	15 (88.2%)	
Lymph nodes	14 (18.9%)	5 (29.4%)	7 (17.5%)	2 (11.8%)	
Histopathologic type					
DC	152 (73.8%)	39 (69.6%)	73 (76%)	40 (74.1%)	0.415 ^b^
Lobular carcinoma	33 (16%)	8 (14.3%)	16 (16.7%)	9 (16.7%)	
Others	21 (10.2%)	9 (16.1%)	7 (7.3%)	5 (9.2%)	
Luminal subtype					
Luminal A like	78 (37.9%)	18 (32.1%)	38 (39.6%)	22 (40.7%)	0.450 ^b^
Luminal B like	114 (55.3%)	33 (58.9%)	50 (52.1%)	31 (57.4%)	
Unknown	14 (6.8%)	5 (8.9%)	8 (8.3%)	1 (1.9%)	
Endocrine resistance					
No	128 (62.1%)	35 (62.5%)	59 (61.5%)	34 (63%)	
Yes	78 (37.9%)	21 (37.5%)	37 (38.5%)	20 (37%)	0.981 ^b^
Primary resistance	20 (25.6%)	7 (33.3%)	8 (21.6%)	5 (25%)	
Secondary resistance	58 (82.8%)	14 (66.7%)	29 (78.4%)	15 (75%)	
Prior chemotherapy					
No	109 (52.9%)	35 (62.5%)	50 (52.1%)	24 (44.4%)	0.161 ^b^
Yes	97 (47.1%)	21 (37.5%)	46 (47.9%)	30 (55.6%)	

Abbreviations: CDK4/6i: cyclin-dependent kinase 4 and 6 inhibitor; ET: endocrine therapy; AI: aromatase inhibitor; IDC: invasive ductal carcinoma; * Mean age at CDK4/6i initiation in years; ^a^: *p* value for Anova test (age at CDK4/6i initiation): ribociclib−palbociclib, *p* = 0.014; ribociclib−abemaciclib and abemaciclib−palbociclib, *p >* 0.05). ^b^: *p* value for chi-square test (menopausal status: abemaciclib−palbociclib and palbociclib−ribociclib, *p <* 0.05; abemaciclib−ribociclib, *p >* 0.05).

**Table 3:** Treatment exposure and patient response data for each CDK4/6i.

Characteristics	Total N (%)	Abemaciclib	Palbociclib	Ribociclib	*p*-Value
Disease progression *					
No	109 (52.9%)	35 (62.5%)	50 (52.1%)	24 (44.4%)	0.161 ^b^
Yes	97 (47.1%)	21 (37.5%)	46 (47.9%)	30 (55.6%)	
Exitus *					
No	151 (73.3%)	42 (75%)	70 (72.9%)	39 (72.2%)	0.941 ^b^
Yes	55 (26.7%)	14 (25%)	26 (27.1%)	15 (27.8%)	
Dose reduction of CDK4/6i					
No	98 (47.6%)	26 (46.4%)	46 (47.9%)	26 (48.1%)	0.980 ^b^
Yes	108 (52.4%)	30 (53.6%)	50 (52.1%)	28 (51.9%)	
Overall response rate	93 (45.1%)	31(51.4%)	35 (36.5%)	27 (50%)	0.132 ^b^
Best response					
Complete response	22 (10.7%)	5 (8.9%)	8 (8.3%)	9 (16.7%)	0.132 ^b^
Partial response	71 (34.5%)	26 (46.4%)	27 (28.1%)	18 (33.3%)	
Stable disease	88 (42.7%)	21 (37.5%)	48 (50%)	19 (35.2%)	
Progression disease	25 (12.1%)	4 (7.1%)	13 (13.5%)	8 (14.8%)	
Best response in premenopausal women					
Complete response	5 (12.5%)	1 (7.7%)	1 (12.5%)	3 (15.8%)	0.280 ^b^
Partial response	18 (45%)	8 (61.5%)	2 (25%)	8 (42.1%)	
Stable disease	14 (35%)	4(30.8%)	5 (62.5%)	5 (26.3%)	
Progression disease	3 (7.5%)	0	0	3 (15.8%)	
Overall response rate in premenopausal women	23 (57.5%)	9 (69.2%)	3 (27.5%)	11 (57.9%)	0.172 ^b^
Time to recurrence					
Time ≤ 12 months	3 (2%)	2 (4.9%)	1 (1.4%)	0 (0.0%)	0.268 ^b^
Time > 12 months	147 (98%)	39 (95.1%)	70 (98.6%)	38 (100%)	

Abbreviations: CDK4/6i: cyclin-dependent kinase 4 and 6 inhibitor; *: Disease progression to CDK4/6i or exitus (42-month cutoff date); ^b^ *p* value for chi-square test.

## Data Availability

Data from this study are available upon request to the corresponding author.
